# Association between sleep parameters and chronic kidney disease: findings from iranian ravansar cohort study

**DOI:** 10.1186/s12882-023-03177-3

**Published:** 2023-05-17

**Authors:** Niloofar Hemati, Farshad Shiri, Farrokhlegha Ahmadi, Farid Najafi, Mehdi Moradinazar, Ebrahim Norouzi, Habibolah Khazaie

**Affiliations:** 1 Sleep Disorders Research Center, Kermanshah University of Medical sciences, Kermanshah, Iran; 2 Department of Inorganic Chemistry, Faculty of Chemistry, Razi University, Kermanshah, Iran; 3 Department of Nephrology, Nephrology Research Center, Tehran University of Medical Sciences, Tehran, Iran; 4 Research Center for Environment Determinants of Health (RCEDH), Health Institute, Kermanshah University of Medical sciences, Kermanshah, Iran

**Keywords:** Sleep medicine, Chronic kidney disease, Sleep duration, Glomerular filtration rate

## Abstract

**Introduction:**

The relationship between sleep duration and chronic kidney disease (CKD) has received relatively little attention in the Kurdish community. Considering the ethnic diversity of Iran and the importance of the Kurdish community, the present study investigated the association between sleep parameters and CKD among a large sample of Iranian-Kurds.

**Methods:**

This cross-sectional study was conducted among 9,766 participants (*M*_age_: 47.33, *SD* = 8.27, 51% female) from the Ravansar Non Communicable Disease (RaNCD) cohort study database. Logistic regression analyses were applied to examine the association between sleep parameters and CKD.

**Results:**

Results showed that prevalence of CKD was detected in 1,058 (10.83%) individuals. Time to fall asleep (*p* = 0.012) and dozing off during the day (*p* = 0.041) were significantly higher in the non-CKD group compared to the CKD group. Daytime napping and dozing off during the day in females with CKD were significantly more than males with CKD. A long sleep duration (> 8 h/day) was associated with 28% (95% CI: 1.05, 1.57) higher odds of CKD compared to normal sleep duration (7 h/d), after adjusting for confounding factors. Participants who experienced leg restlessness had a 32% higher probability of developing CKD than those who did not experience leg restlessness (95% CI: 1.03, 1.69).

**Conclusion:**

Results suggest that sleep duration and leg restlessness may be associated with an increased likelihood of CKD. Consequently, regulating sleep parameters may play a role in improving sleep and preventing CKD.

## Introduction

According to the 2019 Global Disease Burden (GDB), the age-standardized prevalence and death rate for Chronic Kidney Disease (KD) are 8724 and 15.9 per 100,000 people respectively. The percentage change in prevalence and death between 1990 and 2017 was 1.2% and 2.8%, respectively [1]. Thus, given the increasing prevalence of CKD, identifying potential risk factors can be helpful in preventing it.

Previous research showed that sleep parameters may impact CKD. CKD is defined as an estimated glomerular filtration rate (eGFR) < 60 mL/min/1.73 m2. Sleep parameters are defined as self-reported nighttime sleep hours, sleep delays, sleep duration, morning wakeup hours, day time naps, working night shifts, leg restlessness, and use of sleeping pills [2]. Brindle et al. [3] found that a sleep duration between 5 h 20 min and 7 h 6 min per night was associated with a healthy sleep pattern, while more sleep duration demonstrates better sleep health. However, prolonged sleep duration may cause CKD directly through problems with the sympathetic nervous system and angiotensin-aldosterone renin, and/or indirectly through conditions like obesity, type 2 diabetes, and hypertension, which are known CKD risk factors [4, 5]. The association between sleep duration and CVD risk factors may also be connected to devastating changes in kidney function and cardiometabolic disorders [2]. Yet, few studies have examined the relationship between sleep duration, albuminuria, and CKD. Indeed, kidney dysfunction is an important risk factor for cardiovascular disease (CVD) [5].

In their work examining the connection between sleep problems and cardiometabolic conditions, Killick et al. [6] found that sleep deprivation and deficiency can increase endothelial dysfunction, a condition that occurs in CKD. Thus, the prevalence of cardiometabolic disorders is higher in short sleepers than in normal sleepers. Still, very little is known about the impact of long sleep duration on CKD and its associated ailments.

The autonomic nervous system, endothelial function, and metabolism are all factors in CKD development, and regulated by sleep [7], though hypertension is considered the most important risk factor for CKD. Thus, the possibility of reducing blood pressure by altering sleep duration has garnered recent interest. For example, Gangwisch et al. [8] found a significant relationship between short sleep duration and the development of hypertension in cross-sectional and epidemiological studies. However, relatively few studies have examined the relationship between sleep duration and CKD [9–11].

A good night’s sleep is necessary for maintaining hemostatic balance [12]. In some studies, the relationship between sleep and CKD has been examined [9–11]. However, there is still insufficient information available in this regard. For instance, Petrov et al. [11] found a correlation between shorter and longer sleep durations and higher urine albumin-to-creatinine ratios in American adults. Moreover, Petrov et al. (2016) showed that microalbuminuria and a higher glomerular filtration rate (eGFR) were linked to short sleep duration. Yamamoto et al. [13] reported that short sleep (less than 5 h) was associated with a 28% increase in proteinuria (over an average of 2.5 years) among Japanese adults who had no impaired renal function at baseline. In a meta-analysis study, Cheungpasitporn and colleagues [14] showed that short sleep duration was associated with proteinuria, a surrogate marker for kidney disease progression; although the association was not statistically significant.

The present study sought to contribute knowledge about relationship between sleep duration and the development of CKD, particularly Kurdish region. Additionally, there are concerns about poor health care access and utilization in the Kurdish region of Iran [15], including no information about the prevalence of CKD among this population. Therefore, we conducted the present study to investigate the association between the sleep parameters and the CKD among adults in the Kurdish region of Iran.

## Methods

### Study design and populations

**Fig. 1:**
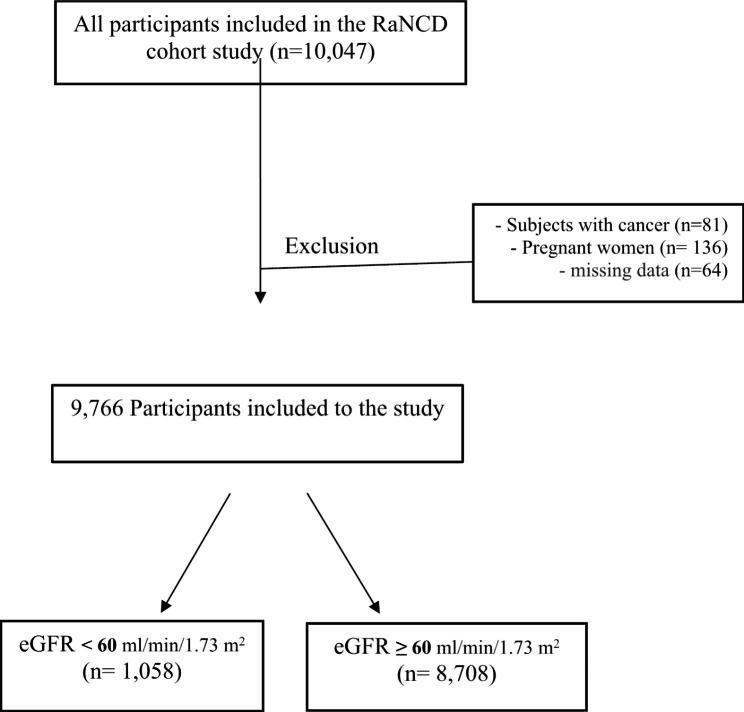
Flow chart of study

This cross-sectional study was conducted using data from the baseline phase of the Ravansar Non-Communicable Disease (RaNCD) cohort study in Ravansar city, Kermanshah province in west of Iran [16] and further information is available at http://persiancohort.com and in the cohort protocol. The RaNCD study is part of the Prospective Epidemiological Research Studies in Iran (PERSIAN). The RaNCD cohort study began in 2014 with the enrollment of 10,047 adults aged 35 to 65 years. The Ethics Committee of Kermanshah University of Medical Sciences approved the study, and all participants provided oral and written informed consent. Participants were excluded from the analyses if they were pregnant, had cancer, or had missing data. Thus, the final sample size for the analysis was 9,766 people (Fig. 1).

### Data collection and measurements

Participants’ basic characteristics, including demographic data (age and gender), socioeconomic status (SES), and lifestyle (smoking, alcohol consumption and physical activity levels) was recorded using the PERSIAN study digital questionnaires with trained experts at the cohort center. Three categories for SES (lowest to highest) were generated by a principal component analysis (PCA) with variables for education, economic well-being, income, and type of residence. Physical activity was measured with a questionnaire about exercise, work, and leisure-related physical activities based on the metabolic equivalent of task (MET)/hours/day. Participants were then classified into three groups of physical activity, including low (24–36.5), moderate (36.6–44.4) and high (≥ 44.5) MET/hours/ day.

Blood urea nitrogen (BUN) and creatinine (Cr) were measured using a Pars Azmoon kit (Pars Azmoon, Tehran, Iran). Body mass index (BMI) and waist-to-hip ratio (WHR) were measured with a bioimpedance Analyzer (BIA) (InBody 770 Biospace, Korea).

Type 2 diabetes mellitus (T2DM) was defined as fasting blood sugar (FBS) levels equal to or higher than 126 mg/dL and/or treatment with anti-diabetic medications. Hypertension was defined as a systolic blood pressure (SBP) ≥ 140 mmHg and diastolic blood pressure (DBP) ≥ 90 mmHg or participants taking medication for hypertension. Cardiovascular diseases (CVDs) were defined as a history of ischemic heart disease (IHD), heart failure and angina, stroke, myocardial infarction (MI), and/or the current use of medication for CVDs.

### **Glomerular filtration rate calculation**

The eGFR was calculated with the Modification of Diet in Renal Disease (MDRD) according to the following equation [17]:

Men: 175 × Serum Cr ^− 1.154^ × age^− 0.203^.

Women: 175 × Serum Cr ^− 1.154^ × age^− 0.203^ × 0.742.

Decreased kidney function was defined as eGFR < 60 mL/min/1.73m^2^ according to the Kidney Disease Improving Global Outcomes criteria for CKD.

### Assessment of sleep parameters

In population-based studies, sleep parameters are usually self-reported and not assessed objectively using devices such as polysomnography [18]. Consistent with this practice, data on self-reported habitual sleep parameters in this study were collected using a standardized questionnaire, measuring nighttime sleep hours, time to fall asleep, sleep duration, morning wakeup hours, daytime naps, working night shift, leg restlessness, use of sleeping pills use, and dozing off during the day. Sleep duration was converted into four categories: very short (< 5 h), short (5–6 h), normal (7–9 h), and long (> 9 h) [19, 20]. Day time napping was defined as taking daily naps (15 to 60 min) three or more times per week. Working night shift was defined as at least 6 h of work between 9PM and 6AM. Leg restlessness was defined as having a restless feeling in the legs while asleep. Use of sleeping pills more than 2 times per week was measured using a 4-point Likert-type scale, ranging from 0 (never) to 3 (always).

### Statistical analysis

Descriptive data were reported as mean scores and standard deviations for continuous variables and counts and percentages for categorical variables. An independent samples *t* test and chi-square test were used to compare baseline characteristics between the two groups (CKD and non-CKD). One-way ANOVA and chi-square tests were used to compare the baseline characteristics and sleep parameters. The association between sleep parameters and CKD development was assessed by logistic regression modeling. A crude model was reviewed as well as one that was adjusted for potential confounds, such as age, gender, smoking status, alcohol intake, and physical activity. P values of 0.05 and lower were regarded as significant. Version 14.2 of STATA software was used for all the analysis.

## Results

Participants’ basic characteristics according to their eGFR levels are shown in Table 1. A total of 9,766 participants including (4703 men and 5063 women) were examined.

**Table 1:** Baseline characteristics of participants according to glomerular filtration rate value (n = 9,766)

Baseline characteristics	Total	CKD (eGFR < 60 ml/min/1.73 m^2^)		P value*	Non- CKD (eGFR ≥ 60 ml/min/1.73 m^2^)		P value*	P value **
		Men	Women		Men	Women		
**Age**								
**35–50 year**	6341 (64.93)	76 (32.48)	358 (43.45)	0.003	3065 (68.58)	2842 (67.04)	0.124	< 0.001
**51–65 years**	3425 (35.07)	158 (67.52)	466 (56.55)		1404 (31.42)	1397 (32.96)		
**Physical activity (Met/h/day)**								
**Light**	2956 (30.27)	87 (37.18)	226 (27.43)	< 0.001	1548 (34.64)	1095 (25.83)	< 0.001	
**Moderate**	4625 (47.36)	78 14.31)	467 (56.67)		1359 (33.31)	2721 (64.19)		0.004
**Vigorous**	2185 (22.37)	69 (29.49)	131 (15.90)		1562 (34.95)	423 (9398)		
**Socioeconomic status**								
**1(lowest)**	3222 (33.01)	76 (32.48)	550 (66.75)	< 0.001	852 (19.07)	1744 (41.17)	< 0.001	
**2**	3268 (33.48)	76 (32.48)	204 (24.76)		1459 (32.65)	1529 (36.10)		< 0.001
**3(Highest)**	3272 (33.52)	82 (35.04)	70 (8.50)		2157 (48.28)	963 (22.37)		
**Current smoker n (%)**	1138 (11.71)	35 (15.22)	29 (45.31)	< 0.001	1003 (93.39)	71 (1.68)	< 0.001	< 0.001
**Alcohol drinking n (%)**	478 (4.89)	14 (5.98)	0	< 0.001	462 (10.34)	2 (0.05)	< 0.001	< 0.001
**T2DM n (%)**	845 (8.65)	39 (16.67)	97 (11.77)	0.048	344 (7.70)	365 (8.61)	0.120	< 0.001
**CVD n (%)**	1658 (16.98)	71 (30.34)	283 (34.34)	0.252	511 (11.43)	793 (18.71)	< 0.001	< 0.001
**Hypertension n (%)**	1543 (15.80)	84 (35.90)	212 (25.73)	0.002	610 (13.65)	637 (15.03)	0.066	< 0.001
**Depression n (%)**	308 (3.15)	7 (2.99)	41 (4.98)	0.198	90 (2.01)	170 (4.01)	< 0.001	< 0.001
**Proteinuria (≥ 1+)**	235 (2.36)	13 (5.56)	28 (3.41)	0.135	88 (1.97)	103 (2.43)	0.142	0.001
**BUN (mg/dl)**	13.62 ± 4.21	17.32 ± 6.58	13.97 ± 5.70	< 0.001	14.77 ± 3.83	12.13 ± 3.51	< 0.001	< 0.001
**Creatinine (mg/dl)**	0.99 ± 0.22	1.53 ± 0.61	1.14 ± 0.33	< 0.001	1.06 ± 0.13	0.87 ± 0.10	< 0.001	< 0.001
**ALT (mg/dl)**	24.92 ± 14.78	26.70 ± 14.74	20.75 ± 13.01	< 0.001	29.40 ± 16.48	20.91 ± 11.45	< 0.001	< 0.001
**AST (mg/dl)**	21.42 ± 9.10	24.19 ± 10.91	21.38 ± 7.95	< 0.001	23.10 ± 9.91	19.51 ± 7.73	< 0.001	0.026
**GGT (mg/dl)**	24.73 ± 19.87	27.63 ± 16.35	22.97 ± 18.19	< 0.001	28.14 ± 20.82	21.31 ± 18.67	< 0.001	0.206
**BMI (kg/m** ^**2**^ **)**	27.49 ± 4.63	26.46 ± 3.81	27.84 ± 4.94	< 0.001	26.34 ± 4.06	28.68 ± 4.86	< 0.001	0.650
**WHR**	0.94 ± 0.10	0.94 ± 0.06	0.94 ± 0.05	0.484	0.93 ± 0.06	0.94 ± 0.05	< 0.001	0.113
**Energy intake (calorie/d)**	2651.91 ± 953.90	2703.37 ± 854.26	2063.30 ± 741.11	< 0.001	3049.81 ± 958.81	2343.98 ± 806.89	< 0.001	< 0.001
**Sleep duration (h/day)**	7.10 ± 1.23	7.02 ± 1.39	7.16 ± 1.31	0.154	6.99 ± 1.19	7.18 ± 1.22	< 0.001	0.221
**Time to fall asleep (Min)**	28.52 ± 17.22	22.83 ± 9.33	36.86 ± 21.97	< 0.001	22.11 ± 12.73	31.95 ± 21.19	< 0.001	0.012
**Daytime napping n (%)**	6166 (63.14)	169 (72.22)	526 (63.83)	0.017	2895 (64.78)	2576 (60.77)	< 0.001	0.068
**Nap duration (Min)**	66.21 ± 46.02	73.01 ± 52.31	65.41 ± 43.81	0.061	65.86 ± 48.31	66.31 ± 43.19	0.719	0.523
**Night shift work n (%)**	1155 (11.83)	48 (20.51)	5 (0.61)	< 0.001	1073 (24.01)	29 (0.68)	< 0.001	< 0.001
**Leg Restlessness n (%)**	517 (5.29)	7 (2.99)	36 (4.37)	0.637	199 (4.45)	275 (6.49)	< 0.001	0.104
**Dozing off during the day n (%)**	3253 (33.31)	72 (30.77)	310 (37.62)	0.054	1497 (33.50)	1374 (32.41)	0.282	0.041
**Use sleeping pills n (%)**	211 (2.16)	6 (2.56)	23 (2.79)	0.851	66 (1.48)	116 (2.74)	< 0.001	0.169
**Night sleep (hours)**	11.31 ± 1.70	15.24 ± 10.33	12.91 ± 10.89	0.003	11.15 ± 11.08	9.23 ± 10.85	< 0.001	0.003
**Morning wakeup (hours)**	6.62 ± 1.20	6.00 ± 1.22	6.41 ± 1.18	< 0.001	6.47 ± 1.18	6.87 ± 1.17	< 0.001	< 0.001

Data are shown mean ± SD for continuous variables and yes/no data based on numbers and percent [n (%)] categorical variables.

*P- value was obtained t-test and Chi – square test for different between male and women

** P- value was obtained t-test and Chi – square test for different between two groups of CKD and non-CKD

Abbreviation: BMI: Body mass index, BUN: Blood Urea Nitrogen, CVD: Cardiovascular diseases, T2DM:Type 2 diabetes mellitus; WHR: Waist hip ratio

**Fig. 2:**
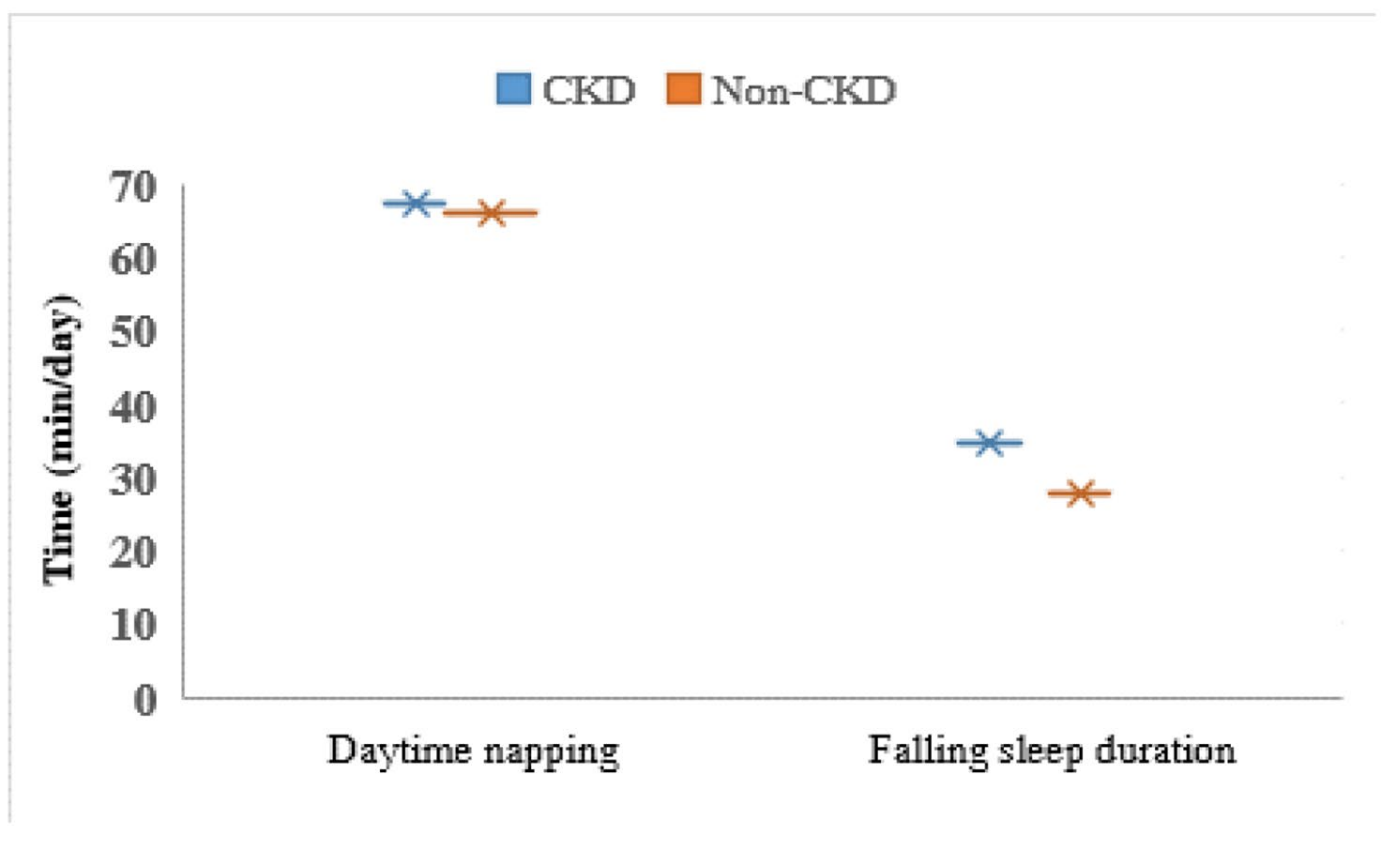
Daytime napping and falling sleep duration in CKD and non-CKD participants

Results showed that the mean age was 47.33 years, with 6,341 (64.93%) aged 35–50 years and 3,425 (35.07%) aged 51–65 years. The prevalence of CKD (eGFR < 60 ml/min/1.73 m^2^) was 10.83% (1,058 participants). The mean sleep duration was 7.10 ± 1.23 h/d in total populations. Low physical activity among individuals with CKD was significantly higher than the non-CKD. Moreover, the level of high physical activity in men of both groups was significantly higher than women (*p* < 0.001). The prevalence of T2DM, hypertension and CVDs among individuals with CKD was significantly higher compared to non-CKD (*p* < 0.001). Time to first fall asleep was longer in the non-CKD group than in those with CKD (*p* = 0.012), and time spent dozing off during the day was longer in the non-CKD group (*p* = 0.041). Results of the independent *t* test showed a significant difference between males and females for sleep duration (*t*(9764) = 7.13, *p* = 0.001) and CKD (*t*(9764) = 18.25, *p* = 0.001). Moreover, daytime napping and dozing off during the day in women with CKD was significantly more likely than among men with CKD. The length of daytime naps and time to fall asleep is shown in Fig. 2 for participants with and without CKD.

Table 2 displays the fundamental traits of the participants by category of sleep duration.

**Table 2:** Baseline characteristics of participants according to sleep duration (n = 9,766)

Baseline characteristics	Sleep duration (h/d)				P value*
	< 4	4–6	6–8	> 8	
**Age**					
**35–50 year**	104 (1.64)	1324 (20.88)	4053 (63.92)	860 (13.56)	< 0.001
**51–65 years**	99 (2.89)	898 (26.22)	2067 (60.35)	361 (10.54)	
**Gender**					
**Men**	106 (2.25)	1117 (23.75)	3021 (64.24)	459 (9.76)	< 0.001
**Women**	97 (1.92)	1105 (21.83)	3099 (61.21)	762 (15.05)	
**Physical activity (Met/h/day)**					
**Light**	53 (26.11)	569 (25.61)	1804 (29.48)	530 (43.41)	< 0.001
**Moderate**	85 (41.87)	1064 (47.88)	2961 (48.38)	515 (42.18)	
**Vigorous**	65 (32.02)	589 (26.51)	1355 (22.14)	176 (14.41)	
**Current smoker n (%)**	26 (12.94)	268 (12.10)	726 (11.93)	118 (9.73)	0.004
**Alcohol drinking n (%)**	14 (6.90)	117 (5.27)	301 (4.92)	46 (3.77)	0.125
**T2DM n (%)**	24 (11.82)	243 (10.94)	481 (7.86)	97 (7.94)	< 0.001
**CVD n (%)**	45 (22.17)	453 (20.39)	988 (16.14)	172 (14.09)	< 0.001
**Hypertension n (%)**	34 (16.75)	386 (17.37)	968 (15.82)	155 (12.69)	0.004
**Depression n (%)**	8 (3.94)	55 (2.48)	179 (2.92)	66 (5.41)	< 0.001
**Proteinuria (≥ 1+)**	4 (1.97)	42 (1.89)	157 (2.57)	29 (2.38)	0.340
**Creatinine (mg/dl)**	1.01 ± 0.16	0.99 ± 0.25	0.99 ± 0.18	0.99 ± 0.31	0.910
**BUN (mg/dl)**	13.99 ± 4.34	13.79 ± 4.46	13.64 ± 4.04	13.11 ± 4.51	< 0.001
**eGFR (ml/min/1.73 m** ^**2**^ **)**	75.04 ± 14.35	76.40 ± 14.24	76.43 ± 13.87	74.84 ± 14.03	0.002
**ALT (mg/dl)**	25.72 ± 18.92	24.99 ± 14.10	25.19 ± 15.10	23.32 ± 13.63	< 0.001
**AST (mg/dl)**	22.63 ± 20.95	21.36 ± 8.10	21.46 ± 8.49	21.08 ± 10.26	0.134
**GGT (mg/dl)**	25.13 ± 16.20	25.42 ± 21.69	24.73 ± 19.27	23.40 ± 19.85	0.040
**BMI (kg/m** ^**2**^ **)**	27.63 ± 5.23	27.69 ± 4.67	27.48 ± 4.60	27.11 ± 4.58	0.005
**WHR**	0.94 ± 0.10	0.94 ± 0.06	0.94 ± 0.06	0.94 ± 0.06	0.703
**Energy intake (kcal/d)**	2814.31 ± 1195.91	2702.18 ± 1006.23	2646.63 ± 29.13	2559.59 ± 924.84	< 0.001

*P- value was obtained Chi – square and one-way ANOVA tests

Abbreviation: BMI: Body mass index, BUN: Blood urea nitrogen, CVD: Cardiovascular diseases, T2DM:Type 2 diabetes mellitus; WHR: Waist hip ratio

**Fig. 3:**
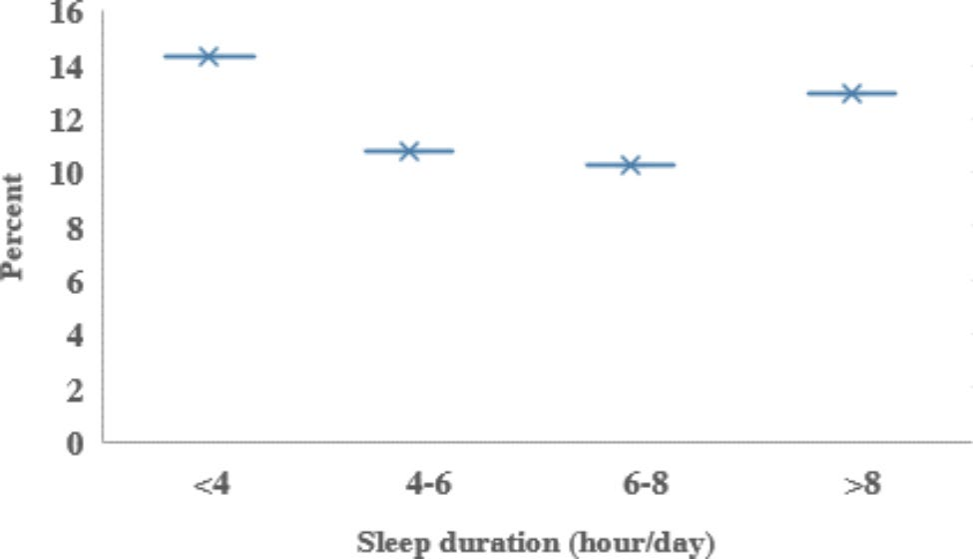
Sleep duration status in participants with CKD

The percentage of participants who slept less than 4 h or 4–6 h was higher in the age group of 51–65 years. Long sleep duration (> 8 h/d) was higher in men than women. In individuals who slept more than 8 h/d, 43% engaged in light physical activity and 14% in vigorous physical activity (*p* < 0.001). Nevertheless, the highest prevalence of depression was observed among participants who slept too long (> 8 h/day). The eGFR levels were lower in individuals with < 4 and > 8 h/day sleep duration (P = 0.002). Participants with CKD’s sleep duration status are shown in Fig. 3.

Table 3 displays results from the logistic regression analysis examining the relationship between sleep and CKD. Compared to normal sleep duration (6–8 h/d), long sleep duration (> 8 h/d) was significantly associated with higher odds for CKD (OR:1.30, 95% CI:1.10, 1.56). Thus, the longer the sleep, the higher the incidence of CKD. After adjusting for age, gender, smoking, alcohol consumption and physical activity long sleep duration (> 8 h/d) was also associated with 28% higher odds of CKD (OR: 1.28; 95% CI: 1.05, 1.57) compared to normal sleep duration (6–8 h/d). However, by controlling gender, the relationship between sleep duration and CKD was not significant (*p* ˃0.05). Time to fall asleep and dozing off during the day was associated with higher odds for CKD, although this relationship was not statistically significant. Participants with leg restlessness had 32% higher odds of CKD than those without leg restlessness (OR: 1.32; 95% CI: 1.03, 1.69), after adjusting for confounding factors.

**Table 3:** Association between the sleep parameters with the chronic kidney disease

Sleep parameters	Crude		Model 1		Model 2	
	OR (95% CI)*	P value*	OR (95% CI)	P value	OR (95% CI)	P value
**Sleep duration**						
6–8 h/d	Ref.		Ref.		Ref.	
< 4 h/d	1.45 (0.97, 2.17)	0.070	1.18 (0.77, 1.81)	0.436	1.13 (0.73, 1.74)	0.581
4–6 h/d	1.05 (0.90, 1.23)	0.517	0.93 (0.79, 1.10)	0.377	0.92 (0.78, 1.10)	0.307
> 8 h/d	**1.30 (1.10, 1.56)**	**0.007**	**1.28 (1.06, 1.56)**	**0.014**	**1.28 (1.05, 1.57)**	**0.013**
**Time to fall asleep (Min)**	**1.01 (1.01, 1.03)**	**0.013**	0.99 (0.99, 1.01)	0.178	0.99 (0.99, 1.01)	0.224
**Day time nap n (%)**	1.13 (0.99, 1.30)	0.523	1.01 (0.99, 1.03)	0.703	1.01 (0.99, 1.03)	0.570
**Night shift work n (%)**	**0.36 (0.27, 0.48)**	**< 0.001**	0.98 (0.72, 1.34)	0.901	0.93 (0.68, 1.29)	0.702
**Leg Restlessness n (%)**	1.14 (0.90, 1.46)	**0.268**	**1.34 (1.05, 1.71)**	**0.020**	**1.32 (1.03, 1.69)**	**0.029**
**Dozing off n (%)**	**1.14 (1.01, 1.31)**	**0.041**	0.93 (0.81, 1.07)	0.363	0.91 (0.79, 1.05)	0.203
**Use sleeping pills n (%)**	1.32 (0.89, 1.96)	0.170	0.93 (0.61, 1.41)	0.726	0.92 (0.61, 1.40)	0.718

* Odds Ratio (95% Confidence Interval) was obtained logistic regression models

Model 1: Adjusted for age and gender

Model 2: Adjusted for age, gender, smoking status, alcohol intake and physical activity

The other results were related to whether sleep duration might predict CKD. A logistic regression was performed. The logistic regression output indicated the overall regression was not statistically significant (OR: 1.03; 95% CI: 0.98, 1.09, *p* = 0.22), suggesting that sleep parameters cannot significantly predict variance in CKD.

## Discussion

The present study demonstrated that sleep duration and leg restlessness may be associated with an increased likelihood of CKD among Iranian adults in the Kurdish region. After adjusting for confounding factors, time of falling sleep, daytime napping, dozing off during the day and use of sleeping pills had no statistically significant association with CKD.

Previous studies that examined the association between sleep duration and kidney function have presented similar and inconsistent results with the results of the present study. Ye et al. [21] found the association between sleep duration and kidney health outcomes was U-shaped, and daytime napping also had a negative effect on kidney health. According to a cohort study in Taiwan [19] lower sleep quality and duration scores were associated with a higher possibility of CKD development. Moreover, experienced difficulty falling asleep and use of sleeping pills were associated with an increased risk of CKD. While, in our study, data on sleep duration was collected by self-reported questionnaires, the subjective-objective discrepancies in sleep duration need to be considered. Alfano et al. [22] found that subjective reports exhibited low and non-significant correspondence with objective sleep patterns and problems measured by an Actigraphy. Therefore, these discrepancies should be considered in interpret the present result.

Chronic Renal Insufficiency Cohort (CRIC) findings have shown that shorter sleep duration and later sleep timing were associated with lower eGFR in US adults [23]. However, a cohort study conducted in Japan [24] showed no association between sleep duration and kidney functioning. Other studies suggest there may be a bidirectional relationship between sleep duration and kidney function. The proposed mechanism is that increasing the activity of the sympathetic nervous system in the body can lead to fragmented sleep and conversely, fragmented sleep activates the sympathetic nervous system. This bidirectional relationship can create a circadian rhythm and kidney dysfunction reinforcing each other [23]. Furthermore, scientific evidence suggests that long sleep duration is associated with subclinical inflammation and increased arterial stiffness, which can lead to decreased kidney function over time [25–27]. In general, sleep disturbances are common among patients with chronic kidney disease. In line with our study, D’Onofrio et al. (2017) found a relationship between kidney diseases and sleep quality and suggested an interdisciplinary approach to the issue, combining the expertise of nephrologists and psychiatrists for people with CKD [28].

Another factor that may mediate the association between long sleep duration and kidney dysfunction is sedentary lifestyle [29]. In the present study, low physical activity was significantly higher in participants with eGFR < 60 ml/min/1.73 m^2^. However after adjusting for physical activity the regression models did not reduce the association between sleep duration and CKD. Therefore, the hypothesis that physical activity could influence the relationship between sleep duration and CKD needs further investigation.

Still, underlying pathways could be involved in the association between sleep duration and kidney function. Sleep disorders have been observed in obesity, decreased insulin sensitivity, and hypertension. In turn, these conditions can cause kidney dysfunction through abnormal production of adipokines and hormones, hemodynamic changes, and eventually glomerular hypertension [30–32]. Indeed, several conditions have been proposed in connecting sleep duration with cardiometabolic dysfunction including oxidative stress, inflammation, endothelial dysfunction, and insulin resistance [33, 34]. For instance, sleep durations (both short and long) lead to increased risk for cardiometabolic dysfunction and metabolic syndrome and these can increase plasma glucose, dyslipidemia, and blood pressure which increases the risk for developing CKD.

One population-based study of Chinese middle-aged people showed that compared to those who did not nap, daytime napping had a negative effect on kidney health. Moreover, the odds of microalbuminuria (an early sign of vascular damage) were 30–57% higher in those who daytime napped [21]. Furthermore, daytime napping has been positively associated with albuminuria in Japanese populations [35]. In contrast, Lin et al. (2018) showed that napping was not associated with CKD [36]. According to the results of the present study, daytime napping was higher in CKD patients and women than in non-CKD and men, although was not statistically significant. The discrepancies in these results could possibly be due to participant differences in race lifestyle, and health. For example, Non-Hispanic Blacks and Mexican Americans are at higher risk for albuminuria [37, 38]. Therefore, future research should consider the level of risk for CKD across different races and ethnicities.

The present study had two primary strengths. First, the large sample size available for analysis. Second, to our knowledge, it is the first study examining the prevalence of and possible factors related to CKD in the Kurdish-Iranian community. However, there were some limitations. The design of the study was cross-sectional; therefore, it was not possible to determine a cause-and-effect relationship. Though several potential confounds were controlled in the analysis, residual confounding effects may be present, such as hormonal effects and genetic characteristics. Moreover, sleep parameters were measured subjectively with self-reported measures, rather than objectively. Still, whether measured objectively or subjectively, some studies have shown inconsistent results regarding the connection between sleep paramters and CKD. Therefore, the combination of these measurement methods could be beneficial in future research [7]. Randomized clinical trials and longitudinal studies on different populations and ethnicities are also recommended, to confirm the association between sleep parameters and CKD.

## Conclusion

The key findings of the present study were that long sleep duration and leg restlessness are significantly associated with greater odds of CKD. Therefore, regulating sleep duration could be a strategy to improve sleep and prevent CKD. It is recommended that physicians consider asking their patients to modify their sleep duration to potentially may help to prevent the occurrence of CKD. However, population-based longitudinal studies on different ethnic/racial groups are needed to better assess the association between sleep parameters and kidney function.

## Data Availability

The data analyzed in the study are available from the corresponding author on reasonable request.
